# Neuroprotective Proteins in Hypoxia-stressed Astrocyte-Derived Extracellular Vesicles

**DOI:** 10.2174/011570159X359837250611052037

**Published:** 2025-06-19

**Authors:** Berenice N. Bernal-Vicente, Isaac Ponce, Manuel Santos-Gutiérrez, Emmanuel Ríos-Castro, Luis B. Tovar-y-Romo

**Affiliations:** 1 Department of Molecular Neuropathology, Instituto de Fisiología Celular, Universidad Nacional Autónoma de México, Mexico City, 04510, Mexico;; 2 Department of Earth and Planetary Science, Weizmann Institute of Science, Herzl 234, Rehovot, 7610001, Israel;; 3 Unidad de Genómica, Proteómica y Metabolómica, LaNSE, Cento de Investigaciones y Estudios Avanzados, Mexico City, 07360, Mexico

**Keywords:** Astrocyte, hypoxia, extracellular vesicles, proteomics, EV cargo, neuroprotection, stroke

## Abstract

**Background:**

Advances in mass spectrometry-based proteomic analysis have generated extensive protein data from cells involved in neurodegenerative diseases. The field of neuroproteomics is expanding to include the study of extracellular vesicles (EVs) to identify potential biomarkers for disease prevention and endogenous factors involved in neuroprotection.

**Methods:**

In this study, rat cortical astrocytes in normoxia were cultured under normoxic conditions and subsequently exposed to hypoxia. Astrocyte-derived EVs released into the supernatant were collected separately from both conditions. Label-free mass spectrometry-based proteomics was then performed to assess the effects of hypoxia on the EV protein cargo. A meta-analysis comparing the results with previously published EV proteomic datasets was also conducted.

**Results:**

This study revealed a differential expression of 83 upregulated proteins under hypoxic conditions and 61 downregulated proteins under normoxic conditions, highlighting the protective protein signatures elicited by astrocytes. The dataset has been deposited in the ProteomeXchange Consortium with the identified PXD050160.

**Conclusion:**

The present study makes a novel contribution by employing proteomic techniques to characterize the protein cargo of EVs isolated from primary rat astrocytes. This approach enables a more refined analysis of astrocyte-specific intercellular signaling under hypoxic conditions and provides valuable insights into the roles of astrocytes in maintaining brain homeostasis and contributing to pathological processes.

## 1 INTRODUCTION

In recent years, research in neuroproteomics has advanced notably in studying EVs derived from brain cells. These vesicles, including exosomes and microvesicles, are key mediators of intercellular communication, facilitating the transfer of biochemical information between cells in the nervous system and impacting brain communication functions [1]. The comprehensive analysis of proteins expressed in a biological system has become essential for understanding the complexity of brain EVs. Advanced proteomics techniques have enabled identifying and characterizing proteins within these vesicles, revealing cell signaling mechanisms and providing insights into brain pathophysiology [2-4].

The molecular responses of astrocytes under hypoxic conditions constitute a crucial focus in neurobiology research, shedding light on the complex cellular mechanisms that occur during oxygen deprivation. Hypoxia, a condition often resulting from events such as ischemic stroke or traumatic brain injury, triggers a cascade of molecular adaptations in astrocytes, critical for maintaining neuronal health and function. Understanding these adaptive responses is of great clinical importance, as it can provide insights into the cellular and molecular pathways that support neuronal survival and recovery following oxygen deprivation. In this sense, astrocytes exhibit unique transcriptomic responses under hypoxia [5, 6]. These responses involve the upregulation of hypoxia-inducible factors (HIFs) and subsequent downstream effectors, which play a fundamental role in cellular survival pathways, angiogenesis, and metabolic reprogramming [7, 8]. Changes in protein expression regularly follow transcriptomic adaptations and can be mirrored in the proteomic cargo of EVs [9, 10].

It was previously reported that EVs produced by astrocytes under normoxic conditions or following a hypoxic challenge help to increase the pace of neurological recovery in experimental stroke [11]. These responses are relatively specific for the parental cell types that produce the EVs; for example, EVs shed by proliferating neuronal precursors are somewhat limited in their potential for neurological recovery [12]. Thus, astrocyte-derived EVs mediate important mechanisms for spontaneous recovery, a phenomenon typically occurring within the first few months following a stroke that represents the brain's natural ability to reorganize and adjust after injury [13].

The present research is focused on characterizing astrocyte-derived EV protein cargo, with a growing interest in their therapeutic potential and their function as neurological disease biomarkers.

## 2 MATERIALS AND METHODS

### 2.1 Animals

The animals used in this study were bred at the Instituto de Fisiología Celular, in the institute's animal facility, which operates in full compliance with Mexican regulations, including NOM-062-ZOO-1999 for the ethical care and use of laboratory animals and NOM-087-ECOL-SSA1-2002 for the management of biological waste.

Wistar rats were used as experimental models for this study. Firstly, adult male rats (270-300 g body weight; n=3) were utilized for intracerebral administration of EVs. These animals were housed under standard conditions, including a 12-hour light/dark cycle, a controlled temperature of 23°C, and a relative humidity of 45%. Animals had *ad libitum* access to a standard diet and water. All experimental procedures involving animals were carried out under current Mexican legislation for using and caring for laboratory animals (NOM-062-ZOO-1999) with the Institutional Animal Care and Use Committee (CICUAL-IFC-LTR93-16) approval.

In a different set of experiments, we employed rat neonates (P1-P2; n=20), as described next, following protocols described previously [11].

### 2.2 Astrocyte Tissue Culture

For tissue culture, primary astrocytes were isolated from the cortices of 1-2-day-old Wistar rats. Briefly, brain tissue was enzymatically digested with trypsin and mechanically dissociated in Hanks’ solution. Cells were cultured in poly-D-lysine-coated flasks with DMEM/F-12 medium supplemented with 10% FBS. After 24 hours, cultures were shaken overnight at 37°C to remove non-adherent cells. The remaining population was ~98% GFAP-positive astrocytes. These experiments were conducted using cells between passages 3 and 5.

### 2.3 EVs Isolation and Characterization

Conditioned media from three independent astrocyte cultures per condition were used to harvest EVs under two experimental conditions: normoxia (NxEV) incubated for 48 hours and hypoxia (HxEV) incubated for 6 hours in a hypoxia chamber (Stemcell, Stemcell Technologies Inc., Canada) with a 100% N_2_ atmosphere at 37°C, followed by a 42-hour recovery under normoxia.

To collect exosomes secreted exclusively by astrocytes, conditioned media from cells cultured with 0.5% exosome-free Fetal Bovine Serum (FBS) was collected and filtered through a 220 nm pore membrane. The medium was ultracentrifuged at 50,000 ´ g for 30 minutes, followed by 100,000 ´ g for 70 minutes. The pellet was resuspended in 100 µl of Phosphate-Buffered Saline (PBS). Nanoparticle Tracking Analysis (NTA) measurement was performed by Nanosight (NS300 Malvern Panalytical UK). For mass spectrometry, EVs were resuspended in 100 μL of low salt binding buffer (LSB) Tris-NaCl pH 7.5.

EVs were characterized by transmission electron microscopy. For this, 2 µL of an EV suspension were placed on a light-discharged copper/carbon grid and stained for 1 minute with 2% uranyl formate. EVs were observed at an accelerating voltage of 80 keV with a JEOL-JEM-1200 transmission electron microscope.

### 2.4 Brain Administration of Astrocyte-derived EVs

In a different set of experiments, EVs, pre-stained with PKH26 (Sigma-Aldrich), were administered by intracerebroventricular injection (i.c.v.) in the brains of young adult 8-week-old male Wistar rats in a total volume of 2 µL, equivalent to a total protein content of 400 ng. Experiments were performed in triplicate. Experimental procedures were approved by the Institutional Animal Care and Use Committee (CICUAL-IFC-LTR93-16) and reported in compliance with the Updated Animal Research: Reporting *in vivo* Experiments (ARRIVE 2.0) guidelines [14].

After 24 hours of EVs administration, the rats were transcardially perfused, the brains were collected and sliced into 40 µm coronal sections that were posteriorly immunolabeled for GFAP (1:500, Sigma; G3893), revealed by Alexa Fluor 488 (conjugated anti-mouse antibody; 1:500, ThermoFisher), and stained with 4′,6-diamidino-2-phenylindole (DAPI, ThermoFisher; 1:10000). All tissue samples were observed by LSM 800 microscope (Zeiss, JENA, GER).

### 2.5 Sample Preparation for Mass Spectrometry

EVs suspensions were processed with a protease inhibitor cocktail (RocheDiagnostics GmbH) and sonicated twice in a 10-pulse cycle over 3 min in a Branson Sonifier 250. The concentration of protein was determined using the 2-D Quant kit (Cytiva, Marlborough, MA, USA), and samples were depleted from albumin using a ProteoMiner protein enrichment kit (BioRad Hercules, CA, USA). Pull-downs of each condition (normoxia and hypoxia) were performed in triplicates. The eluted proteins were precipitated using a methanol/chloroform 4:1 ratio (v:v) and digested using the iST Sample Preparation Kit (PreOmics, Munich, GER), according to manufacturer specifications. Briefly, to the protein pellets, 50 µL of “lysis” reagent was added, and the samples were heated in a thermo block (Eppendorff, Hamburg, GER) for 10 minutes at 95°C. Afterward, the samples were coldly sonicated using a BioRuptor^®^ Pico (Diagenode, Liège, Belgium) for 20 cycles (each cycle runs for 30 seconds on/off).

Subsequently, the samples were enzymatically digested at 37°C for two hours using 50 µL of a mixture of Lys-C/Trypsin (“Digest” reagent). The tryptic peptides were desalted in a C18 iST cartridge and dried on SpeedVac (ThermoFisher Scientific, Waltham, MA, USA). Finally, 15 µL of “LC-Load” reagent were added to the dry peptides for resuspension and stored at -80°C until LC-MS analysis.

### 2.6 Mass Spectrometry-based Proteomics

Quantitative proteomic analysis was performed in a QTOF mass spectrometer Synapt G2-S*i* coupled with a Nano Acquity M-Class (Waters; Milford, MA, USA), according to a previously published protocol with some modifications [15, 16]. Briefly, tryptic peptides were separated by triplicate on an HSS T3 C18 column (Waters, Milford, MA). Mobile phases were prepared according to the following composition: mobile phase A, 0.1% formic acid (FA) in H_2_O, and mobile phase B, 0.1% FA in acetonitrile (ACN) also; the LC gradient was designed using a flow of 400 nL·min-1 as follows: 0 minutes 7% B, 121.49 minutes 40% B, 123.15 to 126.46 minutes 85% B, and 129 to 130 minutes 7% B. Finally, the column temperature was programmed at 45°C. Full-Scan DIA through High-Definition Multiplexed MS/MS (HDMS^E^) mode and nanoelectrospray ionization (nanoESI) was used for data acquisition.

The tune page for ionization source control was set with the following parameters: 30 V in the sampling cone and the source offset 2.75 kV in the capillary emitters, 70°C for the source temperature, 0.5 bar and 150 L·h-1 for the nanoflow gas and for the purge gas flow respectively. LC-MS chromatograms were acquired with a scan time of 500 ms, comprising a range of m/z 50-2000 in positive mode. High-energy chromatograms were obtained by collision-induced dissociation (CID) fragmentation using an energy ramp from 19 to 55 eV inside the transfer cell. The mass spectrometer was calibrated using [Glu^1^]-fibrinopeptide fragments ([M+2H]^2+^ = 785.84261) at less than one ppm across all MS/MS measurements.

### 2.7 MS-Data Analysis

The mass spectra contained in the *.raw files were quantified as previously reported [17], using Progenesis QI for Proteomics software v3.0.3 (Waters Corp, Milford MA, USA) utilizing a target decoy strategy against a concatenated (reverse sense) *Rattus norvegicus* *.fasta database (downloaded from UniProt, 29,971 protein sequences) [18] to estimate all false positives in the analysis. The parameters used during the database search were set as follows: trypsin as the cleavage enzyme fixed modification: cysteine carbamidomethylation; variable modification: methionine oxidation, C-terminal amidation, also, glutamine and asparagine deamidation, serine, threonine, and tyrosine phosphorylation and, only one missed cleavage was allowed for peptide identification. Besides, minimum fragment ion matches: two and five per peptide and protein, respectively; minimum peptide matches per protein: one peptide and fragment tolerance were set to automatic, and a false discovery rate (FDR) ≤ 4%.

False-positive identifications, annotated as reversed proteins, and proteins with only one peptide identified were eliminated from further analysis. After annotating the proteins against the rat proteome, we manually annotated each protein that did not have an updated Uniprot Accession or Protein Symbol assigned. Two proteins (A0A096MJ53 and A0A0G2JVY3) were classified as uncharacterized. We analyzed the resulting dataset using custom scripts in Python 3.11 (Supplementary Table **1**: Proteomics_data_set_curated.xlsx. in Filtered proteins). Differential expression analysis was performed using a two-sample t-test, and *p*-value correction (FDR) was applied using the Benjamini-Hochberg procedure. The protein expression heatmap and volcano plot were generated using the seaborn package. Significantly, the differentially expressed proteins with an FDR < 0.05 were used for functional annotation analysis, performed using the Database for Annotation, Visualization, and Integrated Discovery (DAVID) Bioinformatics Resources Web Customer Service (https://david.ncifcrf.gov/) with default settings in a semi-automated script available upon request.

Functional annotation analysis was performed using GO terms, Reactome, and KEGG pathways. Differentially expressed proteins were also analyzed using STRING (Szklarczyk *et al*., 2019) for functional analysis (confidence score of 0.7), cluster analysis, and text mining from articles. For cluster analysis, the Markov Cluster Algorithm with an inflation parameter of 2.5 was used. The proteomics data have been deposited to the ProteomeXchange Consortium *via* the PRIDE [19] partner repository with the dataset identifier PXD050160.

### 2.8 Data Mapping

The database of DIA was plotted as a heatmap and volcano plots using R software to screen for differentially expressed proteins (fold-change>1.20 or <0.83, *p* <0.05). Common differentially expressed proteins were selected using R software. The differential protein expression (DPE), gene ontology (GO), and Kyoto Encyclopedia Genes and Genomes (KEGG) analysis were analyzed by Database for Annotation, Visualization, and Integrated Discovery (DAVID) Bioinformatics Resources (https://david.ncifcrf.gov/) and complemented with GProfiler web server. Q-value < 0.5 indicated statistical significance. Statistical analysis values ​​presented in this study are expressed as the mean ± SD. The Simpson index was used for meta-analyses. All samples were carried out in triplicate or more. The error probability *p* = < 0.5 was considered statistically significant.

## 3 RESULTS

### 3.1 EVs Isolation and Characterization from Cultured Primary Astrocytes

**Fig. (1):**
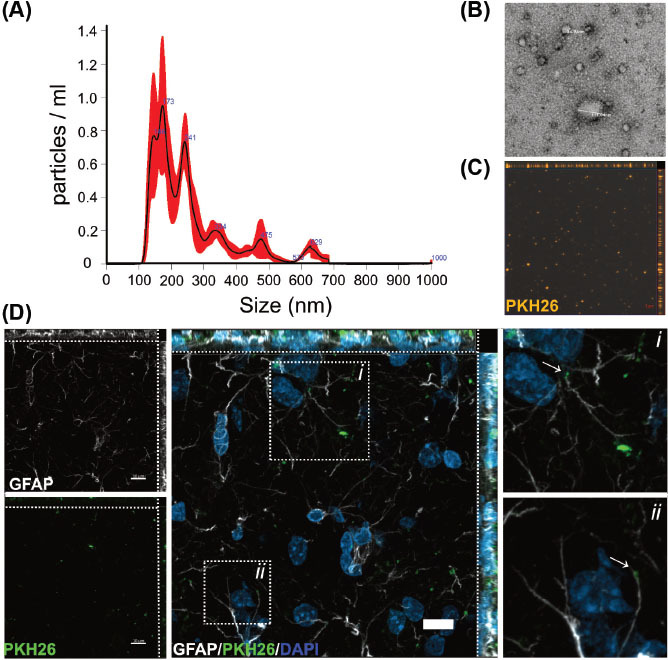
Characterization of EVs produced by primary cortical astrocytes in culture (**A**). Size distribution of particles in suspensions by nanoparticle tracking analysis of EVs isolated from cultured cortical astrocytes. (**B**). Transmission electron microscopy micrographs of EVs isolated from astrocyte cultures. Vesicles were visualized by negative staining with uranyl formate on copper/carbon-coated grids. (**C**). PKH26-stained EVs (orange) were observed under a confocal microscope. (**D**). Distribution of EVs stained with PKH26 (green) injected icv into the brain of rats within the striatum. EVs internalized in astrocytes (GFAP; white). Dotted squares demark regions where EVs are located; images on the left are magnifications of those regions; the images are maximum projections of a z-stack of 20 optical slices showing the orthogonal planes. Nuclei are stained with DAPI (blue); scale bars, 10 µm.

Primary astrocytes were obtained from P1-2 Wistar rats following our previously described protocol [11]. In a set of experiments, the cells were subjected to hypoxia for six hours and reintroduced to standard culture conditions for 42 hours. Conditioned media with 0.5% FBS-depleted from EVs were collected from a total volume of 150 mL. The size and quantity of EVs isolated were determined with NTA, showing a peak size within the typical range of exosomes for both conditions: normoxia at 161 ± 73.6 nm and hypoxia at 140.1 ± 40.2 nm (mean ± SD), the overall concentration of these suspensions was 5.80^008^ ± 4.07^007^ particles/mL for normoxia and 3.15^008^ ± 2.70^07^ particles/mL for hypoxia (Fig. **1A**). We harvested a uniform population of small vesicles exhibiting characteristic morphology of exosomes under both conditions, as shown by TEM (Fig. **1B**). Additionally, a small number of EVs with larger sizes were also observed in the sample. This suggests that vesicles subjected to hypoxia are smaller in size and are present at a lower concentration compared to those in the normoxic control, indicating that oxygen deprivation directly influences the secretion of these vesicles by the cell.

### 3.2 Exogenous Astrocyte-derived EVs are Internalized by Astrocytes

We conducted an *in vivo* administration of EVs in the brains of three adult male Wistar rats. PKH26-stained EVs (Fig. **1C**) were injected into the lateral ventricle of anesthetized rats using a glass microcapillary pipette. The animals were euthanized 24 hours post-injection, and the brains were fixed in 4% PFA. Coronal sections of 40 µm thickness were then obtained for immunofluorescence analysis. As shown in Fig. (**1D**), 24 hours after EV delivery to the lateral ventricle, stained vesicles were observed in the striatum and somatosensory cortex of both cerebral hemispheres, internalized in target cells such as astrocytes, suggesting that these vesicles carry homing molecular cues.

### 3.3 Astrocyte-derived EVs Proteomics

We conducted a proteomic profile by LC-MS/MS from samples obtained from astrocyte-derived EVs subjected to normoxic conditions or six hours of hypoxia. EV samples were produced in triplicates and collected in a low salt binding buffer (LSB). EV samples were purified through albumin depletion using the ProteoMiner kit (Thermo Fisher Scientific) and analysed by LC-MS/MS. Qualitative and quantitative analyses were performed by Synapt G2-S*i* mass spectrometry using a label-free approach. We detected 210 proteins in both conditions. Differential expression analyses resulted in 83 proteins upregulated under hypoxia and 61 proteins downregulated in normoxia. (Supplementary Table **1**: Curated proteomics dataset; FDR<0.05 upregulated and FDR<0.05 downregulated). All differentially expressed proteins (DEP) are represented in Fig. (**2A**).

Among the DEP, it was found that Histone H2A (gene H2afy) and Serine/threonine-protein kinase (gene Dclk2) were among the most upregulated proteins in hypoxia samples. Other important proteins upregulated in hypoxia include Laminin, Elongation factor 1-delta (Eef1d), Heat shock protein 70 (Hsp-70), Annexin V (Anxa5), Complement component 3 (C3), Nucleophosmin (Npm1), Tubulin alpha-1b chain (Tuba1b), Synergin, Major vault protein (MVP), Desmin, CD81, Glutathione S-transferase, Heat shock protein 90 (HSP90-beta), Tubulin beta-6 chain (Tubb6), Core histone macro-H2A (H2afy), Coagulation factor XIII A (F13a1), Protein transport protein SEC23 (gene Sec23a), and Serine/threonine-protein kinase (Dclk2).

**Fig. (2):**
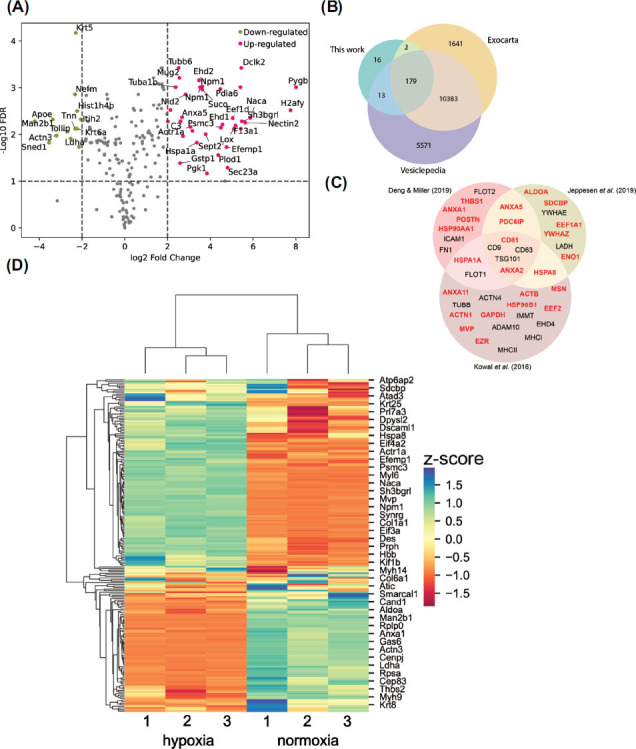
Label-free quantitative LC-MS-based proteomics from astrocyte-derived EVs under normoxia and hypoxia. (**A**). Volcano plot shows differentially expressed proteins (DEPs). Green dots to the left depict downregulated proteins in normoxia, and pink dots to the right show upregulated proteins in hypoxia as regards normoxia (**B**). Venn's diagram of EV protein cargo compared to the reported proteins in ExoCarta and Vesiclepedia (**C**). Venn's diagram of EV protein cargo shows canonical markers expressed in the proteins identified in this study marked in red, compared to three different publications (Kowal *et al*. 2016, Jappensen *et al*. 2019 and Den & Miller 2019). (**D**). Heatmap of 42 proteins identified from the proteomic profiling of astrocyte-derived EVs in normoxic and hypoxic conditions. n=3.

Conversely, Apolipoprotein E (ApoE), Alpha-mannosidase (Man2b1), and Keratin type II cytoskeletal 5 (Krt5) were the most significantly downregulated proteins in normoxia (Fig. **2A**). Other proteins that were also downregulated in the normoxia profile include Histone 4 (Hist1h4b), Tenascin N (Tnn), Endoplasmin, Clathrin, Neurofilament medium polypeptide (Nefm), Periostin (POSTN), Galectin, DEAD-box polypeptide, Nidogen-1, Gelsolin, Moesin, Insulin responsive sequence DNA binding protein-1 (Sned1), Toll-interacting protein (Tollip), L-lactate dehydrogenase (Ldha), Lactadherin, and Keratin type II cytoskeletal 5 (Krt5).

Of special interest, we identified the brain form of glycogen phosphorylase (Pygb), which, along with glycogen synthase (GS), plays a key role in determining the glycogenolysis flux in the brain following an ischemic event. Glycogen mobilization may enhance the production of NADPH and glutathione *via* the pentose phosphate pathway (PPP), which in turn can reduce reactive oxygen species (ROS) levels during reperfusion. Overexpression of glycogen phosphorylase (GP) has been shown to induce neuroprotection by decreasing infarct volume 72 hours after reperfusion [20].

This study confirmed the origin of EVs in the dataset by comparing the astrocyte-derived EVs proteome with the publicly available databases ExoCarta (consulted on 22^nd^ January 2024) and Vesiclepedia (accessed on 22^nd^ January 2024) (Supplementary Table **1**) Curated proteomics dataset; exocarta/vesiclepedia). It was discovered that 194 of our 21013 proteins had been previously detected in EVs (Fig. **2B**). Interestingly, we identified 16 proteins that had not been previously reported to be expressed in EVs, including AP2A2, a component of the clathrin-mediated vesicle transport and endocytosis complex. AP2A2 has been implicated in exocytosis in synaptic terminals [21].

As well, a bioinformatic analysis of astrocyte-derived EV proteins from three published datasets [10, 22, 23] shows the canonical markers of EVs expressed in our dataset with proteins marked in red that are also present in those publications (Fig. **2C**) (Supplementary Table **1**: Curated proteomics dataset; exosome markers). Then, 42 of the most significant genes were used to identify the proteins highly expressed in both groups and to generate a heatmap (Fig. **2D**).

Among the proteins with the highest relative expression in the normoxia control group were Fructose-bisphosphate aldolase A (Aldoa), Cullin-associated NEDD8-dissociated protein 1 (Cand1), Annexin A1 (Anxa1), Growth arrest-specific protein 6 (Gas6), L-lactate dehydrogenase A chain (Ldha), Small ribosomal subunit protein uS2 (Rpsa), and Thrombospondin 2 (Thbs2) (Fig. **2D**). In contrast, the hypoxia group showed much higher expression of Prolactin (Prl7a3), Eukaryotic initiation factor 4A-II (Eif4a2), EGF-containing fibulin-like extracellular matrix protein 1 (Efemp1), 26S proteasome regulatory subunit 6A (Psmc3), Nucleophosmin (Npm1), and Desmin (Des) compared to the normoxia group (Fig. **2D**).

Next, the protein markers of EVs were searched as previously established in the literature [10, 22, 23]. We found 24 protein markers out of 40 different proteins determined to be EV markers, including Alix (Pdcd6ip), a protein previously reported in the primary and cell-line 14 cortical astrocytes [24], Syntenin-1 (Sdcbp), Annexin A2 (Anxa2), CD81 (Cd81), Heat shock protein HSP 90 (HSP90) and Heat shock protein 70 (HSP70) that has been used to purified or characterized exosomes [22]. As expected, many of these markers, classified as canonical markers for EVs [22], were present in these experiments. Most recently, other markers such as Annexin I, Annexin II, Annexin V, Thrombospondin (TSP1), and Periostin (POSTN) have been proposed to be markers for EVs due to their appearance in different kinds of cells and biofluids [23].

Syntenin-1 has been considered to be a marker exclusively from exosomes [10], and some specific markers for microvesicles, such as Annexin A2 and Annexin V, while Annexin I, II, and V, previously considered specific for microvesicles [22], can be expressed by exosomes. Thus, our proteome profile contains a heterogeneous composition of proteins related to exosomes and microvesicle markers (marked in black) that other authors have already proposed.

Some exclusive proteins were also observed, such as profilin-1, phosphoglycerate mutase, calcitonin, and 14-3-3 protein beta/alpha, widely produced in the brain by different cells. These proteins function in various cellular processes in neurons and are considered biomarkers of neurological disorders characterized by extensive destruction of neurons in the brain and stroke-like episodes [25, 26].

### 3.4 Astrocyte-derived EV Protein Cargo Contains Cues for Neuroprotection

**Fig. (3):**
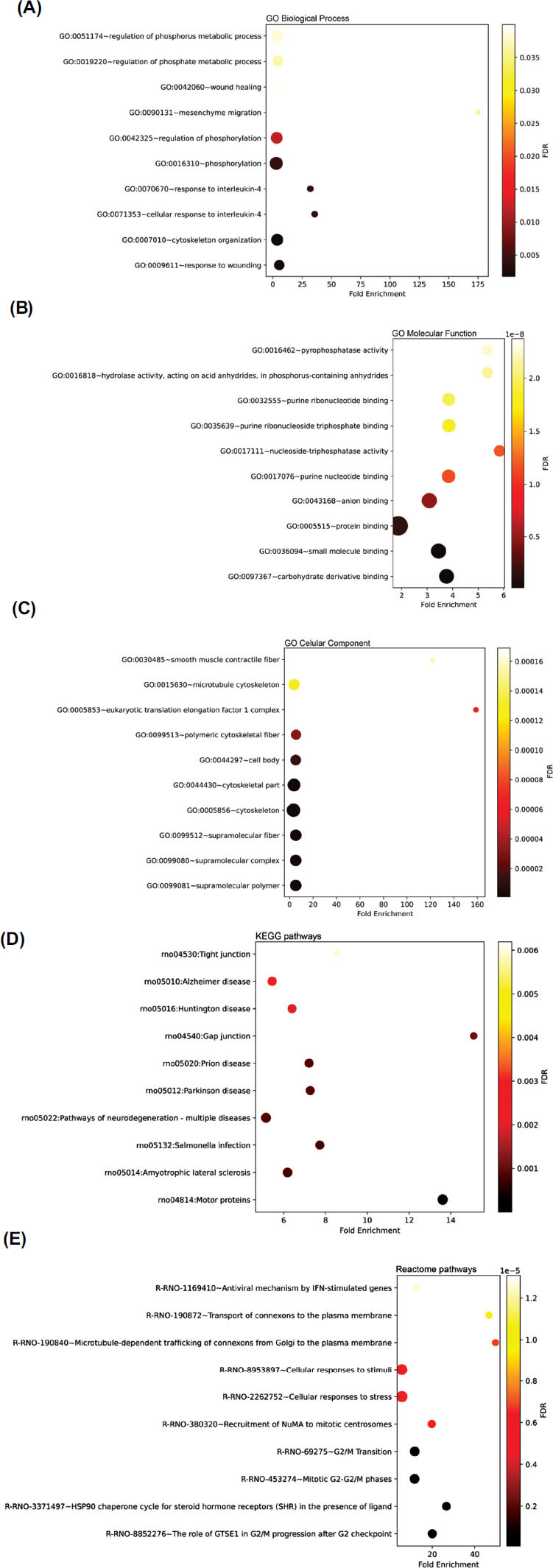
Bioinformatic analysis of the proteomic profile of astrocyte-derived EVs under hypoxia and normoxia. The top ten biological processes (**A**), molecular function (**B**), and cellular component (**C**) of hypoxia- Gene Ontology (GO) analyses identified upregulated proteins. (**D**) Kyoto Encyclopedia of Genes and Genomes (KEGG) annotation database top ten processes in upregulated proteins in hypoxia. (**E**) Reactome pathway annotation of the top ten processes in upregulated proteins in hypoxia. In each case, the plots show the fold enrichment of upregulated proteins and color codes for the false discovery rate.

A bioinformatic analysis was performed with filtered MS-profile data using the Gene Ontology (GO) website https://www.geneontology.org/, categorizing data into biological processes (BP; Fig. **3A**), molecular functions (MF; Fig. **3B**) and cellular components (CC; Fig. **3C**). GO enrichment analysis showed that the cellular response to interleukin-4 (GO:0071353) and phosphorylation (GO:0016310) had the most significant biological processes in the DEP that were upregulated under hypoxic conditions. These were followed by cytoskeleton organization (GO:0007010) and response to wounding (GO:0009611). For molecular functions, the most significantly enriched genes were related to the nucleoside-triphosphatase activity (GO:0017111), with the most significant number of genes associated with carbohydrate derivative binding (GO:0097367) and small molecule binding (GO:0036094). The eukaryotic translation elongation factor 1 complex was the most enriched gene set in cellular components.

Conversely, in the DEP from the downregulated normoxia control group, GO enrichment showed that oxygen transporter activity (GO:0005344) was the most significant molecular function, while the highest number of genes was associated with protein binding (GO:0005515). Additionally, supramolecular fiber organization (GO:0097435) was the most enriched biological process, followed by cellular component biogenesis (GO:0044085) and cellular component assembly (GO:0022607), which involved the most significant number of genes. All GO representative terms are in the Supplementary Data.

The Kyoto Encyclopedia of Genes and Genomes (KEGG) pathway was employed to explore molecular interactions in upregulated proteins further (Fig. **3D**). This analysis showed that the most enriched genes were involved in the Gap junction pathway (rno04540) and motor proteins (rno04814), as well as pathways related to neurodegeneration and multiple diseases (rno05022) (Fig. **3D**). While in downregulated proteins, the most significant KEGG enrichment was glycolysis and gluconeogenesis (rno00010).

The Reactome analysis of upregulated proteins revealed enrichment of the HSP90 chaperone cycle for steroid hormone receptors (SHR) in the presence of a ligand (R-RNO-3371497) (Fig. **3E**).

**Fig. (4):**
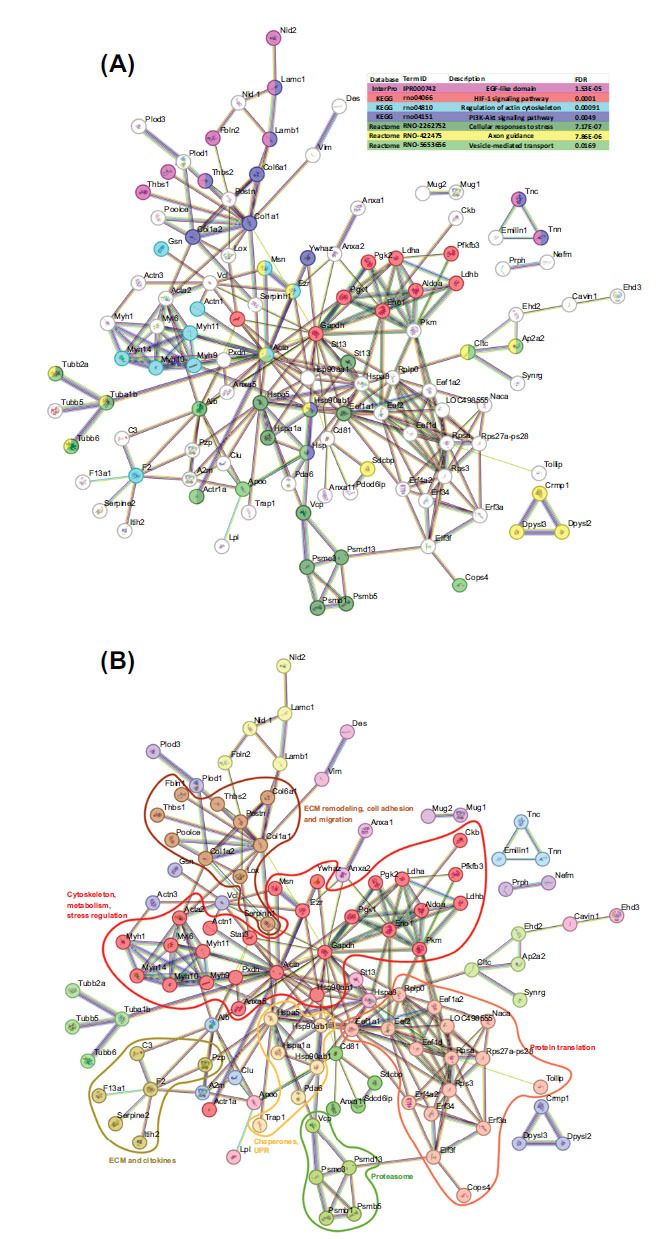
Protein network interactions from the astrocytic EV proteome under hypoxia. Schematic views of the clustering of known and predicted protein interactions according to the STRING database (**A**) and functional associations (**B**). Each node represents a protein, and each color edge represents an interaction. Only interactions with the medium confidence score (FDR) are shown. Interactions include physical and functional associations.

Clustering analysis in STRING identified several protein interaction. The largest interaction cluster, comprising 27 proteins, mainly included proteins involved in cytoskeleton organization and stability, metabolic activity, chaperones, and stress response proteins. Additionally, another interaction cluster with 16 proteins was associated with protein translation, including translation factors, ribosomal proteins, and other related functions such as Naca, which is involved in the early stages of protein folding and quality control. Other smaller clusters, consisting of 10, 6, and 5 proteins, contained proteins related to extracellular matrix remodeling, cell adhesion, cell migration, cytokines, immune-related proteins, proteins involved in the unfolded protein response (UPR), and components of the proteasomal degradation pathway (Fig. **4A**).

This study also evaluated several functional associations: glycolytic enzymatic pathways (red nodes, right), proteasome-related proteins (light green nodes, below), ribosomal translation factors (clear and salmon nodes), molecular chaperone protein family (yellow nodes in the middle), including Hsp90 (Hsp90ab1, Hsp90aa1, Hspa5) which promotes the protection of the proteome from stress, folding, transport, cell cycle, and signal transduction (Fig. **4B**). Additionally, transmembrane proteins were identified related to EVs markers (green nodes) and structural proteins (brown, red, and lilac nodes on the left). DEPs were used as input for STRING, and further functional analysis identified several enriched pathways such as the EGF-like domain, HIF-1 signaling pathway, regulation of actin cytoskeleton, PI3K-Akt signaling pathway, cellular responses to stress, axon guidance, and vesicle-mediated transport. In hypoxia or stroke, these enriched pathways reveal comprehensive cellular reactions aimed at survival and adaptation to an oxygen-deprived environment. Activation of the HIF-1 signaling pathway, a hallmark of hypoxic conditions, orchestrates a cascade of events to promote cell survival, angiogenesis, and metabolic adaptation [27]. Concurrently, cellular stress response pathways are mobilized to counteract the detrimental effects of oxygen deprivation, mitigating oxidative stress and protein misfolding [28]. The upregulation of the PI3K-Akt signaling pathway supports cell survival and metabolic reprogramming, potentially contributing to neuroprotection against ischemic injury. Axon guidance pathways and vesicle-mediated transport mechanisms may play roles in neuronal remodeling and synaptic plasticity following stroke, facilitating recovery and functional restoration.

In summary, the proteomic profile of EVs reveals that hypoxic conditions affect key proteins involved in glycolysis and gluconeogenesis pathways, ribosomal proteins, cell structural components, and the proteasome pathway.

### 3.5 Meta-analysis

**Fig. (5):**
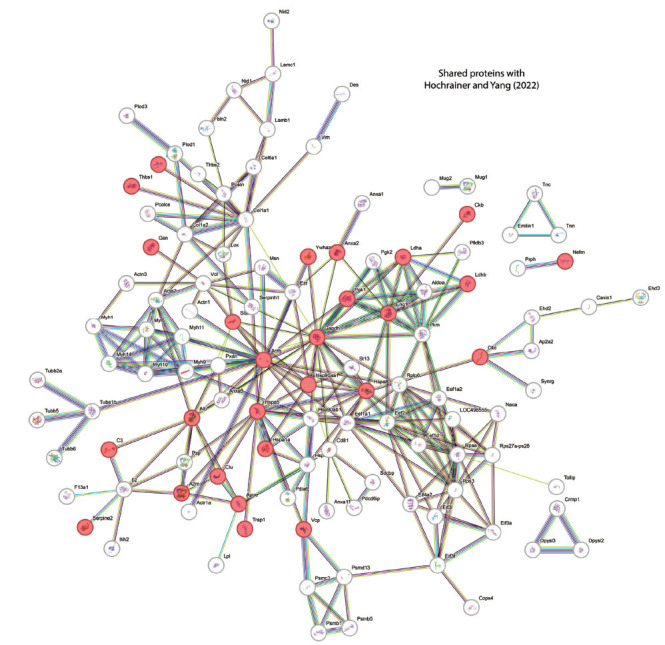
Bioinformatic analysis of astrocyte-derived EVs protein content. STRING analysis of the shared proteins identified in this study with the previously reported meta-analysis by Hochrainer and Yang 2022.

With text mining, we identified a meta-analysis [29], where the authors analyzed 51 studies utilizing proteomics from animal stroke models, mainly from tissue and cerebrospinal fluid from rodents. Our data shares 31 proteins with their dataset, including some proteins that could serve as biomarkers for differential diagnosis and prognosis, such as APOE and STAT3 (Fig. **5**). These proteins are associated with recovery after stroke by contributing to myelination and oligodendrogenesis and mediating anti-inflammatory processes in the ischemic brain [30]; whereas HSP90 reduction was found to be correlated with a more severe injury, two HSP90 isoforms were found upregulated in our dataset (Hsp90ab1, 6.60 fold change and Hsp90aa1, 1.55 fold change). Complement C3 was upregulated in cerebrospinal fluid, while 14-3-3 protein zeta/delta also suggested a high correlation of EV proteins with those obtained from various biofluids or CSF (Fig. **4**).

In this context, upregulated DEPs were identified in hypoxia, suggesting changes in complement, coagulation factors, translational regulation, and protein phosphorylation, indicating heightened responses to hypoxic stress. One of these upregulated proteins is the 14-3-3 protein, which is widely produced in the brain by different cells, drives many processes, including cell differentiation, cell migration, cell survival, neurite outgrowth, and ion channel regulation in neurons, and is considered a biomarker of neurological disorders characterized by extensive destruction of neurons in the brain and stroke-like episodes [25, 26].

**Fig. (6):**
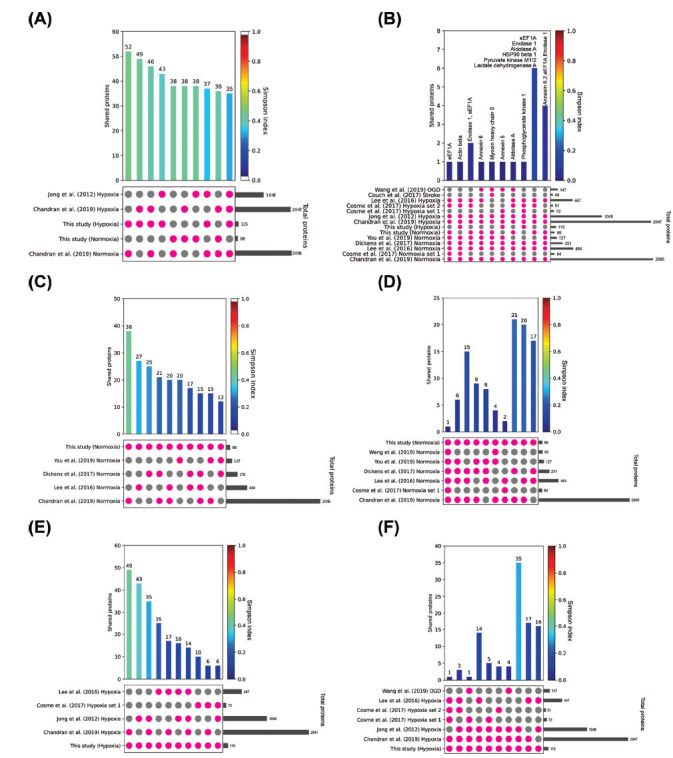
Meta-analysis of proteins from EVs derived from either normoxic (24 non-redundant comparisons) or hypoxic (23 non-redundant comparisons) conditions. (**A**). Top ten comparisons with the highest number of shared proteins. (**B**). Top ten comparisons with the highest number of datasets involved. (**C**). Top ten comparisons of datasets in normoxic conditions with the highest number of shared proteins. (**D**). Top ten comparisons of datasets in normoxic conditions with the highest number of datasets involved. (**E**). Top ten comparisons of datasets in hypoxic conditions with the highest number of shared proteins. (**F**). Top ten comparisons of datasets in hypoxic conditions with the highest number of datasets involved. The bar charts illustrate the number of shared proteins in the datasets highlighted in the column below. Pink dots indicate the datasets included in the comparison, while gray dots represent those excluded. The horizontal gray bar charts show each study's total number of proteins. The bar chart representing the number of shared proteins is colored according to the Simpson diversity index.

To deepen the study of proteins related to hypoxia, oxygen, and glucose deprivation (OGD), and ischemic stroke events and to understand which proteins are associated with astrocyte-derived extracellular vesicles, 16 proteomic datasets were extracted from eight published studies of EV proteomes under normoxic and hypoxic conditions [31-38] and compared them with the data obtained in this study (Fig. **6**). Details of each dataset are shown in Supplementary Table **2** (Meta proteome comparisons). Every possible combination of two or more studies was compared for all the datasets by identifying their common proteins. Given the number of studies, N=16, the total number of possible combinations without repetition is 2N, out of which only those involving two or more datasets are compared. Hence, a total of 2 N - N - 1 = 65,519 comparisons were carried out, each denoted as Ci for i=1,…,65,519. Each comparison Ci possessed the structure of a 4-tuple: 𝐶𝑖 = (𝑟𝑐𝑟𝑟 𝑖𝑙𝑣𝑙𝑙𝑣𝑐𝑐, 𝑐𝑙𝑙𝑙𝑙𝑙 𝑙𝑟𝑙𝑟𝑐𝑖𝑙𝑟, |𝑟𝑐𝑟𝑟 𝑖𝑙𝑣𝑙𝑙𝑣𝑐𝑐|, |𝑐𝑙𝑙𝑙𝑙𝑙 𝑙𝑟𝑙𝑟𝑐𝑖𝑙𝑟|), where 𝑟𝑐𝑟𝑟 𝑖𝑙𝑣𝑙𝑙𝑣𝑐𝑐 is yet another tuple consisting of a combination of the 𝑁 data sets, 𝑐𝑙𝑙𝑙𝑙𝑙 𝑙𝑟𝑙𝑟𝑐𝑖𝑙𝑟 is the intersection of the datasets indicated in 𝑟𝑐𝑟𝑟 𝑖𝑙𝑣𝑙𝑙𝑣𝑐𝑐, and | ⋅ | denote the cardinality of a set or the length of a tuple. The set of comparisons, {𝐶𝑖}2𝑖=𝑁1−𝑁−1, is, further, restricted to those where 𝑟𝑐𝑟𝑟 𝑖𝑙𝑣𝑙𝑙𝑣𝑐𝑐 contains this work's hypoxia and normoxia datasets, with the aim of 20 stressing the findings of the present paper. An estimated 49150 comparisons containing any of our datasets were further used in the downstream analyses. However, this analysis leads to redundancies as regards the information it provides, for example, considering the following two comparisons:

𝐶𝑖1 = ((1,2,3,4),′ 𝑃′, 4,1), 𝐶𝑖2 = ((1,2,3),′ 𝑃′, 3,1), where 𝑖1and 𝑖2 denote two arbitrary comparisons, the tuples (1,2,3) and (1,2,3,4) denote two collections of three and four datasets under comparison, and ′𝑃′ is a given protein. The information provided by 𝐶𝑖2 is redundant, as it adds no extra information concerning what 𝐶𝑖1 provides.

Consequently, redundancies of this sort were removed by taking the disjoint union of tuples in comparisons sharing the same set of common proteins. To find the most relevant dataset comparisons, the sets {𝐶𝑖}2𝑖=𝑁1−𝑁−1 were sorted according to the number of common proteins, *i.e*., |𝑐𝑙𝑙𝑙𝑙𝑙 𝑙𝑟𝑙𝑟𝑐𝑖𝑙𝑟|. The ten comparisons with the highest number of common proteins are shown in Fig. (**6A**) from 251 non-redundant comparisons. To assess the relevance of the comparisons, the Simpson diversity index was computed, which in our analysis would be interpreted as the probability that two or more datasets are of the same type. For each comparison {𝐶𝑖}2𝑖=𝑁1−𝑁−1, the Simpson index, 𝑆𝐼𝑖, is computed as: |𝑟𝑐𝑟𝑟 𝑖𝑙𝑣𝑙𝑙𝑣𝑐𝑐| 𝑆𝐼𝑖 = min |𝑟𝑐𝑟|. This index is, thus, a number ranging from zero to one, where low values ​​indicate a low chance of the sets involved in the comparison being of the same type.

Moreover, the comparisons were examined with the highest number of datasets involved in said comparison. For this purpose, the comparisons { }2𝑖=𝑁1−𝑁−1 are sorted according to the number of sets involved, *i.e*., |𝑟𝑐𝑟𝑟 𝑖𝑙𝑣𝑙𝑙𝑣𝑐𝑐|. The top ten comparisons with the highest number of involved sets are shown in Fig. (**6B**) from 250 non-redundant comparisons.

This study further examined the expression of proteins under normoxia (Fig. **6C**, **D**) and hypoxia (Fig. **6E**, **F**) conditions separately. For this, the same method detailed above was followed, whereby comparisons are selected firstly if they correspond to a dataset of proteins from EVs from either normoxic (24 non-redundant comparisons) or hypoxic (23 non-redundant comparisons) conditions, then to the number of shared proteins and, finally, to the number of datasets sharing the same protein combination. The full list of proteins of each comparison is available in Supplementary Table **2** (Meta proteome comparisons).

35 to 49 proteins were identified in our hypoxic dataset that were also found in previously published studies. The major shared proteins in hypoxic, oxygen-glucose deprivation (OGD), and stroke conditions included β-actin, annexin 5, and phosphoglycerate kinase 1. In normoxic conditions, 12 to 38 proteins from our study were shared across the top 10 comparisons with other datasets. eEF1A, Enolase 1, Aldolase A, 22 HSP90 β1, Pyruvate kinase M1/2, and Lactate dehydrogenase were the most shared proteins between normoxic and hypoxic groups.

## 4 DISCUSSION

Under hypoxic conditions, astrocytes secrete EVs that reduce the damage caused by ischemic stroke [11]. The mechanisms underlying this neuroprotection are not fully understood, although the data in this study suggest that multiple pathways are involved. Studying the transcriptomic landscape of astrocytes under hypoxia is crucial for understanding the complex responses of these glial cells to oxygen deprivation, with significant implications for neurobiology, disease pathology, and therapeutic development. Under hypoxic stress, astrocytes undergo metabolic shifts [39-41], often upregulating glycolytic pathways and modulating lactate production, a critical energy source for neurons [42, 43], and exhibit altered expression of cytokines and chemokines [44, 45]. These molecular responses can exacerbate or mitigate neuroinflammatory outcomes in pathologies like stroke and traumatic brain injury [46-48].

Chronic hypoxic conditions lead to significant epigenetic modifications in astrocytes, including DNA methylation and histone modifications, which alter long-term gene expression patterns [49, 50]. The hypoxia-induced transcriptomic changes in astrocytes also influence their interactions with other glial cells, such as microglia and oligodendrocytes [51, 52]. Under hypoxia, astrocytes can also alter the expression of tight junction proteins and basement membrane components, influencing blood-brain barrier (BBB) ​​permeability [53], and hypoxia-induced changes in astrocytes can affect their role in synaptic plasticity and neurotransmitter homeostasis. For instance, alterations in glutamate transporter expression can significantly affect excitotoxicity in ischemic conditions [54, 55]. Thus, the advent of high-throughput sequencing technologies, such as RNA-seq, has allowed for a more comprehensive and detailed understanding of the molecular shifts triggered within the challenged astrocytes under hypoxic stress to promote recovery [56]. This provides unprecedented resolution in understanding the complexity and dynamics of the molecular regulatory functions of glial cells. Understanding the astrocytic response to hypoxia offers insights into fundamental neurobiological processes and allows for developing targeted therapeutic strategies.

All these gene-regulated events are ultimately reflected in the protein content released in EVs. In this sense, brain EV proteomics holds promise for identifying biomarkers of neurological diseases. Recent studies have examined the proteomic profiles of EVs in experimental models using rats, mice, and human samples, highlighting specific proteins linked to pathological conditions and suggesting potential therapeutic and diagnostic targets [57].

The results presented in this work, along with the publicly available database generated for this study, will enable the identification of molecular effectors that are synthesized in response to hypoxia in astrocytes and may point to cellular pathways involved in tissue resilience that allows brain recovery after an ischemic injury.

## STUDY LIMITATIONS

While this study provides important insights into the proteomic composition of astrocyte-derived EVs under hypoxic conditions, several limitations must be acknowledged that may affect the interpretation and generalizability of the findings.

First, the experiments were conducted exclusively using primary rat cortical astrocytes cultured *in vitro*, which, although highly controlled, cannot fully recapitulate the complex *in vivo* environment of the brain. The absence of other glial or neuronal cell types, as well as the lack of vascular and immune components, may limit the biological relevance of the EV cargo in the broader context of neurovascular interactions.

Second, hypoxia was induced in a single, acute 6-hour exposure, followed by normoxic recovery. While this approach effectively models transient oxygen deprivation, it does not capture the diversity of hypoxic stress seen in different pathological conditions, such as chronic hypoxia or fluctuating oxygen levels following ischemic stroke. The study’s design may therefore overlook temporal dynamics or secondary responses that would occur with prolonged or repeated hypoxic insults.

Third, the functional validation of the identified proteins was not pursued, and their specific roles in neuroprotection or pathology remain speculative, based solely on bioinformatic inference.

Finally, the translational value of the identified protein biomarkers is currently limited, as human tissue or fluid samples have not been examined. While cross-referencing was done with public datasets, the absence of human validation restricts the immediate applicability of these results in clinical biomarker development.

Future work should validate these findings *in vivo* under pathophysiological conditions, explore cell-type comparisons, and assess the relevance of these EV-associated proteins in human biofluids during neurodegenerative or ischemic events.

## CONCLUSION

The present study employed advanced proteomic techniques to comprehensively characterize the protein cargo of EVs isolated from primary rat astrocytes. This targeted approach enables a more precise and systematic analysis of astrocyte-specific intercellular communication mechanisms, particularly under hypoxic conditions that mimic the pathophysiological environment of cerebral ischemia. By isolating EVs directly from astrocytes, the study provides a refined molecular snapshot of how these glial cells participate in brain signaling networks, going beyond traditional models that often overlook the complexity and specificity of EV-mediated communication. The findings offer valuable insights into the dynamic role of astrocytes in maintaining brain homeostasis and modulating responses to injury and disease through EV signaling. This work also underscores the growing recognition of EVs as active conveyors of functional molecules and highlights the potential of astrocyte-derived EVs as central players in neural repair and resilience.

One of the most impactful outcomes of this investigation is the identification of a wide array of previously unreported proteins present in astrocyte-derived EVs. These novel proteins serve as molecular fingerprints of astrocyte activity and represent a valuable resource for deciphering neuron-astrocyte communication, which plays a fundamental role in synaptic regulation, metabolic support, and neurovascular coupling. The proteomic dataset generated here provides a wealth of information on protein-protein interactions and signaling pathways that may strengthen neuroprotective mechanisms and endogenous processes of recovery following cerebral ischemia. Of particular interest is the enrichment of pathways related to oxidative stress response, axonal outgrowth, and synaptic plasticity—key elements of post-ischemic brain repair. These findings advance our understanding of astrocyte biology under ischemic stress and raise the possibility that modulating EV content or release could serve as a therapeutic strategy to enhance brain repair.

Notably, the study provides a structured, searchable database of astrocyte-derived EV proteins, significantly benefiting the researchers in the field. This resource provides a foundation for hypothesis-driven research into the roles of specific EV proteins, as well as systems-level analyses of astrocyte-mediated signaling in both health and disease. Furthermore, this database contributes to the ongoing search for robust, clinically translatable biomarkers of ischemic brain injury—one of the most pressing unmet needs in stroke medicine. By linking astrocyte EV content with distinct phases of ischemic progression and recovery, future studies may be able to identify EV-associated signatures that predict outcomes or guide therapy. Overall, the integrative approach presented in this study, combining cell-type-specific EV isolation, proteomic profiling, and bioinformatic analysis, opens new avenues for exploring the functional relevance of astrocyte-derived EVs and their translational potential in neurovascular disorders.

## Data Availability

The data and supportive information are available within the article.

## LIST OF ABBREVIATIONS

ACN: Acetonitrile
BBB: Blood-brain Barrier
CID: Collision-induced Dissociation
DPE: Differential Protein Expression
EVs: Extracellular Vesicles
FA: Formic Acid
FBS: Fetal Bovine Serum
GO: Gene Ontology
GP: Glycogen Phosphorylase
GS: Glycogen Synthase
HIFs: Hypoxia-inducible Factors
KEGG: Kyoto Encyclopedia of Genes and Genomes
OGD: Oxygen-glucose Deprivation
PBS: Phosphate-buffered Saline
PPP: Pentose Phosphate Pathway
ROS: Reactive Oxygen Species
SHR: Steroid Hormone Receptors
UPR: Unfolded Protein Response

## References

[1] Pantazopoulou M., Lamprokostopoulou A., Karampela D.S., Alexaki A., Delis A., Coens A., Samiotaki M., Kriebardis A.G., Melki R., Pagakis S.N., Stefanis L., Vekrellis K. (2023). Differential intracellular trafficking of extracellular vesicles in microglia and astrocytes.. Cell. Mol. Life Sci..

[2] Khan N.A., Asim M., El-Menyar A., Biswas K.H., Rizoli S., Al-Thani H. (2022). The evolving role of extracellular vesicles (exosomes) as biomarkers in traumatic brain injury: Clinical perspectives and therapeutic implications.. Front. Aging Neurosci..

[3] Lizarraga-Valderrama L.R., Sheridan G.K. (2021). Extracellular vesicles and intercellular communication in the central nervous system.. FEBS Lett..

[4] Vinaiphat A., Sze S.K. (2022). Proteomics for comprehensive characterization of extracellular vesicles in neurodegenerative disease.. Exp. Neurol..

[5] Allen S.P., Seehra R.S., Heath P.R., Hall B.P.C., Bates J., Garwood C.J., Matuszyk M.M., Wharton S.B., Simpson J.E. (2020). Transcriptomic analysis of human astrocytes *in vitro* reveals hypoxia-induced mitochondrial dysfunction, modulation of metabolism, and dysregulation of the immune response.. Int. J. Mol. Sci..

[6] Vangeison G., Rempe D.A. (2009). The Janus-faced effects of hypoxia on astrocyte function.. Neuroscientist.

[7] Baumann J., Tsao C.C., Huang S.F., Gassmann M., Ogunshola O.O. (2021). Astrocyte-specific hypoxia-inducible factor 1 (HIF-1) does not disrupt the endothelial barrier during hypoxia *in vitro*.. Fluids Barriers CNS.

[8] Guo M., Ma X., Feng Y., Han S., Dong Q., Cui M., Zhao Y. (2019). In chronic hypoxia, glucose availability and hypoxic severity dictate the balance between HIF-1 and HIF-2 in astrocytes.. FASEB J..

[9] Schwanhäusser B., Busse D., Li N., Dittmar G., Schuchhardt J., Wolf J., Chen W., Selbach M. (2011). Global quantification of mammalian gene expression control.. Nature.

[10] Kowal J., Arras G., Colombo M., Jouve M., Morath J.P., Primdal-Bengtson B., Dingli F., Loew D., Tkach M., Théry C. (2016). Proteomic comparison defines novel markers to characterize heterogeneous populations of extracellular vesicle subtypes.. Proc. Natl. Acad. Sci. USA.

[11] Heras-Romero Y., Morales-Guadarrama A., Santana-Martínez R., Ponce I., Rincón-Heredia R., Poot-Hernández A.C., Martínez-Moreno A., Urrieta E., Bernal-Vicente B.N., Campero-Romero A.N., Moreno-Castilla P., Greig N.H., Escobar M.L., Concha L., Tovar-y-Romo L.B. (2022). Improved post-stroke spontaneous recovery by astrocytic extracellular vesicles.. Mol. Ther..

[12] Campero-Romero A.N., Real F.H., Santana-Martínez R.A., Molina-Villa T., Aranda C., Ríos-Castro E., Tovar-y-Romo L.B. (2023). Extracellular vesicles from neural progenitor cells promote functional recovery after stroke in mice with pharmacological inhibition of neurogenesis.. Cell Death Discov..

[13] Phan N.V., Rathbun E.M., Ouyang Y., Carmichael S.T., Segura T. (2023). Biology-driven material design for ischaemic stroke repair.. Nat. Rev. Bioeng..

[14] Percie du Sert N., Hurst V., Ahluwalia A., Alam S., Avey M.T., Baker M., Browne W.J., Clark A., Cuthill I.C., Dirnagl U., Emerson M., Garner P., Holgate S.T., Howells D.W., Karp N.A., Lazic S.E., Lidster K., MacCallum C.J., Macleod M., Pearl E.J., Petersen O.H., Rawle F., Reynolds P., Rooney K., Sena E.S., Silberberg S.D., Steckler T., Würbel H. (2020). The ARRIVE guidelines 2.0: Updated guidelines for reporting animal research.. PLoS Biol..

[15] Ríos-Castro E., Souza G.H.M.F., Delgadillo-Álvarez D.M., Ramírez-Reyes L., Torres-Huerta A.L., Velasco-Suárez A., Cruz-Cruz C., Hernández-Hernández J.M., Tapia-Ramírez J. (2020). Quantitative proteomic analysis of MARC-145 cells infected with a mexican porcine reproductive and respiratory syndrome virus strain using a label-free based DIA approach.. J. Am. Soc. Mass Spectrom..

[16] Delgadillo D.M., Céspedes-Cruz A.I., Ríos-Castro E., Maldonado R.M.G., López-Nogueda M., Márquez-Gutiérrez M., Villalobos-Manzo R., Ramírez-Reyes L., Domínguez-Fuentes M., Tapia-Ramírez J. (2022). Differential expression of proteins in an atypical presentation of autoimmune lymphoproliferative syndrome.. Int. J. Mol. Sci..

[17] Li G.Z., Vissers J.P.C., Silva J.C., Golick D., Gorenstein M.V., Geromanos S.J. (2009). Database searching and accounting of multiplexed precursor and product ion spectra from the data independent analysis of simple and complex peptide mixtures.. Proteomics.

[18] Käll L., Storey J.D., MacCoss M.J., Noble W.S. (2008). Assigning significance to peptides identified by tandem mass spectrometry using decoy databases.. J. Proteome Res..

[19] Perez-Riverol Y., Bai J., Bandla C., García-Seisdedos D., Hewapathirana S., Kamatchinathan S., Kundu D.J., Prakash A., Frericks-Zipper A., Eisenacher M., Walzer M., Wang S., Brazma A., Vizcaíno J.A. (2022). The PRIDE database resources in 2022: A hub for mass spectrometry-based proteomics evidences.. Nucleic Acids Res..

[20] Guo H., Fan Z., Wang S., Ma L., Wang J., Yu D., Zhang Z., Wu L., Peng Z., Liu W., Hou W., Cai Y. (2021). Astrocytic A1/A2 paradigm participates in glycogen mobilization mediated neuroprotection on reperfusion injury after ischemic stroke.. J. Neuroinflammation.

[21] Kyung J.W., Cho I.H., Lee S., Song W.K., Ryan T.A., Hoppa M.B., Kim S.H. (2017). Adaptor Protein 2 (AP-2) complex is essential for functional axogenesis in hippocampal neurons.. Sci. Rep..

[22] Jeppesen D.K., Fenix A.M., Franklin J.L., Higginbotham J.N., Zhang Q., Zimmerman L.J., Liebler D.C., Ping J., Liu Q., Evans R., Fissell W.H., Patton J.G., Rome L.H., Burnette D.T., Coffey R.J. (2019). Reassessment of exosome composition.. Cell.

[23] Deng F., Miller J. (2019). A review on protein markers of exosome from different bio-resources and the antibodies used for characterization.. J. Histotechnol..

[24] Morel L., Regan M., Higashimori H., Ng S.K., Esau C., Vidensky S., Rothstein J., Yang Y. (2013). Neuronal exosomal miRNA-dependent translational regulation of astroglial glutamate transporter GLT1.. J. Biol. Chem..

[25] Siman R., Roberts V.L., McNeil E., Dang A., Bavaria J.E., Ramchandren S., McGarvey M. (2008). Biomarker evidence for mild central nervous system injury after surgically-induced circulation arrest.. Brain Res..

[26] Shimada T., Fournier A.E., Yamagata K. (2013). Neuroprotective function of 14-3-3 proteins in neurodegeneration.. BioMed Res. Int..

[27] Semenza G.L. (2012). Hypoxia-inducible factors in physiology and medicine.. Cell.

[28] Hartl F.U., Hayer-Hartl M. (2002). Molecular chaperones in the cytosol: From nascent chain to folded protein.. Science.

[29] Hochrainer K., Yang W. (2022). Stroke proteomics: From discovery to diagnostic and therapeutic applications.. Circ. Res..

[30] Li L., Li R., Zacharek A., Wang F., Landschoot-Ward J., Chopp M., Chen J., Cui X. (2020). ABCA1/ApoE/HDL signaling pathway facilitates myelination and oligodendrogenesis after stroke.. Int. J. Mol. Sci..

[31] Jong D.O.G., Verhaar M.C., Chen Y., Vader P., Gremmels H., Posthuma G., Schiffelers R.M., Gucek M., Balkom V.B.W.M. (2012). Cellular stress conditions are reflected in the protein and RNA content of endothelial cell-derived exosomes.. J. Extracell. Vesicles.

[32] Lee J.Y., Kim E., Choi S.M., Kim D.W., Kim K.P., Lee I., Kim H.S. (2016). Microvesicles from brain-extract—treated mesenchymal stem cells improve neurological functions in a rat model of ischemic stroke.. Sci. Rep..

[33] Couch Y., Akbar N., Davis S., Fischer R., Dickens A.M., Neuhaus A.A., Burgess A.I., Rothwell P.M., Buchan A.M. (2017). Inflammatory stroke extracellular vesicles induce macrophage activation.. Stroke.

[34] Dickens A.M., Tovar-y-Romo L.B., Yoo S.W., Trout A.L., Bae M., Kanmogne M., Megra B., Williams D.W., Witwer K.W., Gacias M., Tabatadze N., Cole R.N., Casaccia P., Berman J.W., Anthony D.C., Haughey N.J. (2017). Astrocyte-shed extracellular vesicles regulate the peripheral leukocyte response to inflammatory brain lesions.. Sci. Signal..

[35] Cosme J., Guo H., Hadipour-Lakmehsari S., Emili A., Gramolini A.O. (2017). Hypoxia-induced changes in the fibroblast secretome, exosome, and whole-cell proteome using cultured, cardiac-derived cells isolated from neonatal mice.. J. Proteome Res..

[36] Chandran I.V., Welinder C., Gonçalves de Oliveira K., Cerezo-Magaña M., Månsson A.S., Johansson M.C., Marko-Varga G., Belting M. (2019). Global extracellular vesicle proteomic signature defines U87-MG glioma cell hypoxic status with potential implications for non-invasive diagnostics.. J. Neurooncol..

[37] Wang X., Wang J., Shi X., Pan C., Liu H., Dong Y., Dong R., Mang J., Xu Z. (2019). Proteomic analyses identify a potential mechanism by which extracellular vesicles aggravate ischemic stroke.. Life Sci..

[38] You Y., Borgmann K., Edara V.V., Stacy S., Ghorpade A., Ikezu T. (2020). Activated human astrocyte-derived extracellular vesicles modulate neuronal uptake, differentiation and firing.. J. Extracell. Vesicles.

[39] Deitmer J.W., Theparambil S.M., Ruminot I., Noor S.I., Becker H.M. (2019). Energy dynamics in the brain: Contributions of astrocytes to metabolism and ph homeostasis.. Front. Neurosci..

[40] Li J., Pan L., Pembroke W.G., Rexach J.E., Godoy M.I., Condro M.C., Alvarado A.G., Harteni M., Chen Y.W., Stiles L., Chen A.Y., Wanner I.B., Yang X., Goldman S.A., Geschwind D.H., Kornblum H.I., Zhang Y. (2021). Conservation and divergence of vulnerability and responses to stressors between human and mouse astrocytes.. Nat. Commun..

[41] Smolič T., Tavčar P., Horvat A., Černe U., Vasle H.A., Tratnjek L., Kreft M.E., Scholz N., Matis M., Petan T., Zorec R., Vardjan N. (2021). Astrocytes in stress accumulate lipid droplets.. Glia.

[42] Yamagata K. (2022). Lactate supply from astrocytes to neurons and its role in ischemic stroke-induced neurodegeneration.. Neuroscience.

[43] Genc S., Kurnaz I.A., Ozilgen M. (2011). Astrocyte - neuron lactate shuttle may boost more ATP supply to the neuron under hypoxic conditions - *in silico* study supported by *in vitro* expression data.. BMC Syst. Biol..

[44] Pantazopoulou V., Jeannot P., Rosberg R., Berg T.J., Pietras A. (2021). Hypoxia-induced reactivity of tumor-associated astrocytes affects glioma cell properties.. Cells.

[45] Mojsilovic-Petrovic J., Callaghan D., Cui H., Dean C., Stanimirovic D.B., Zhang W. (2007). Hypoxia-inducible factor-1 (HIF-1) is involved in the regulation of hypoxia-stimulated expression of monocyte chemoattractant protein-1 (MCP-1/CCL2) and MCP-5 (Ccl12) in astrocytes.. J. Neuroinflammation.

[46] Perriot S., Mathias A., Perriard G., Canales M., Jonkmans N., Merienne N., Meunier C., Kassar E.L., Perrier A.L., Laplaud D.A., Schluep M., Déglon N., Pasquier D.R. (2018). Human induced pluripotent stem cell-derived astrocytes are differentially activated by multiple sclerosis-associated cytokines.. Stem Cell Reports.

[47] Zamanian J.L., Xu L., Foo L.C., Nouri N., Zhou L., Giffard R.G., Barres B.A. (2012). Genomic analysis of reactive astrogliosis.. J. Neurosci..

[48] Khakh B.S., Sofroniew M.V. (2015). Diversity of astrocyte functions] and phenotypes in neural circuits.. Nat. Neurosci..

[49] Neal M., Richardson J.R. (2018). Epigenetic regulation of astrocyte function in neuroinflammation and neurodegeneration.. Biochim. Biophys. Acta Mol. Basis Dis..

[50] Thompson J.W., Dave K.R., Young J.I., Perez-Pinzon M.A. (2013). Ischemic preconditioning alters the epigenetic profile of the brain from ischemic intolerance to ischemic tolerance.. Neurotherapeutics.

[51] Liddelow S.A., Guttenplan K.A., Clarke L.E., Bennett F.C., Bohlen C.J., Schirmer L., Bennett M.L., Münch A.E., Chung W.S., Peterson T.C., Wilton D.K., Frouin A., Napier B.A., Panicker N., Kumar M., Buckwalter M.S., Rowitch D.H., Dawson V.L., Dawson T.M., Stevens B., Barres B.A. (2017). Neurotoxic reactive astrocytes are induced by activated microglia.. Nature.

[52] Wheeler M.A., Jaronen M., Covacu R., Zandee S.E.J., Scalisi G., Rothhammer V., Tjon E.C., Chao C.C., Kenison J.E., Blain M., Rao V.T.S., Hewson P., Barroso A., Gutiérrez-Vázquez C., Prat A., Antel J.P., Hauser R., Quintana F.J. (2019). Environmental control of astrocyte pathogenic activities in cns inflammation.. Cell.

[53] Kaur C., Sivakumar V., Zhang Y., Ling E.A. (2006). Hypoxia-induced astrocytic reaction and increased vascular permeability in the rat cerebellum.. Glia.

[54] Wang Y., Fu A.K.Y., Ip N.Y. (2022). Instructive roles of astrocytes in hippocampal synaptic plasticity: Neuronal activity-dependent regulatory mechanisms.. FEBS J..

[55] Wang Y., Fu W.Y., Cheung K., Hung K.W., Chen C., Geng H., Yung W.H., Qu J.Y., Fu A.K.Y., Ip N.Y. (2021). Astrocyte-secreted IL-33 mediates homeostatic synaptic plasticity in the adult hippocampus.. Proc. Natl. Acad. Sci. USA.

[56] Ito M., Aswendt M., Lee A.G., Ishizaka S., Cao Z., Wang E.H., Levy S.L., Smerin D.L., McNab J.A., Zeineh M., Leuze C., Goubran M., Cheng M.Y., Steinberg G.K. (2018). RNA-sequencing analysis revealed a distinct motor cortex transcriptome in spontaneously recovered mice after stroke.. Stroke.

[57] Quiroz-Baez R., Hernández-Ortega K., Martínez-Martínez E. (2020). Insights into the proteomic profiling of extracellular vesicles for the identification of early biomarkers of neurodegeneration.. Front. Neurol..

